# How gluttonous cell aggregates clear substrates coated with microparticles

**DOI:** 10.1038/s41598-017-15665-2

**Published:** 2017-11-16

**Authors:** Grégory Beaune, Andy Y. W. Lam, Sylvie Dufour, Françoise M. Winnik, Françoise Brochard-Wyart

**Affiliations:** 1 WPI International Center for Materials Nanoarchitectonics (MANA), National Institute for Materials Science (NIMS), 1-1 Namiki, Tsukuba, Ibaraki, 305-0044 Japan; 2 Inserm, U955, Equipe 6, Créteil, 94000 France; 3 Université Paris Est, Faculté de Médecine, Créteil, 94000 France; 4 Department of Chemistry, University of Montreal, CP 6128 Succursale Centre Ville, Montreal, QC H3C3J7 Canada; 5 Department of Chemistry and Faculty of Pharmacy, University of Helsinki, FI, 00014 Helsinki, Finland; 6 Laboratoire Physico Chimie Curie, Institut Curie, PSL Research University, CNRS UMR168, 75005 Paris, France; 7 Sorbonne Université, UPMC Univ Paris 06, 75005 Paris, France

## Abstract

We study the spreading of cell aggregates deposited on adhesive substrates decorated with microparticles (MPs). A cell monolayer expands around the aggregate. The cells on the periphery of the monolayer take up the MPs, clearing the substrate as they progress and forming an aureole of cells filled with MPs. We study the dynamics of spreading and determine the width of the aureole and the level of MP internalization in cells as a function of MP size, composition, and density. From the radius and width of the aureole, we quantify the volume fraction of MPs within the cell, which leads to an easy, fast, and inexpensive measurement of the cell – particle internalization.

## Introduction

The collective migration of cells is essential in many biological and pathological processes, such as embryonic development, wound healing, and cancer metastasis. Coordinated groups of cells can be loosely connected strands, as in the case neurogenesis, 2D-assemblies, such as the cell sheets required to close wounds after injury, or 3D-cell aggregates found in cancer tumors. Recently, we used cellular aggregates as tissue models to describe the dynamics of tissue spreading in the framework of wetting^1^. We study here how cell aggregates interact with an environment polluted by inert particles. This study was prompted by recent reports on the effects of nanoparticles on the migration of single cells and 2D-cell sheets. Single cells migrating on a substrate coated with gold nanoparticles (NP) were shown to “vacuum-clean” the sedimented NPs with their leading edge. They left behind them a trail devoid of particles. As the cells engulf the NPs, their migration properties changed noticeably^2^.

When a cell aggregate is deposited on an adhesive substrate, it spreads by forming a cellular monolayer that progressively expands around the aggregate. We have described the dynamics of spreading by analogy with the spreading of stratified droplets^1^. We adopted this experimental/theoretical approach to assess the effect of particles on the migration of cells from 3D-aggregates. We used aggregates of Ecad-GFP cells, a mouse sarcoma cell line (S180) transfected to express E-cadherin-GFP^3^ and monitored their spreading on a fibronectin-coated substrate covered with microparticles (MP). Three types of MPs were employed: (i) *PsAmine*20*0*, polystyrene particles with amine surface groups (hydrodynamic diameter *d* = 0.8 μm); (ii) *PsCarbo1000*, polystyrene particles with carboxylate surface groups (*d* = 1 μm), and (iii) *SiO*_*2*_ *Carbo1000*, silica particles with carboxylate surface groups (*d* = 1 μm). The spreading of the aggregates was recorded as a function of the time after deposition on a substrate covered with a precisely known MP surface density.

Before analyzing the spreading of aggregates on MP-coated substrates, we recall the dynamics of an aggregate spreading on a bare substrate. As a cell aggregate is deposited on an adhesive (fibronectin) substrate, it spreads by forming a cellular monolayer, the precursor film, that progressively expands around the aggregate. The dynamics of spreading of the precursor film^4^ results from the balance between the driving forces *S* due to the motile cells on the periphery of the film, and the friction forces associated with two types of flow: (i) the permeation corresponding to the entry of cells from the aggregates into the film and (ii) the slippage as the film expands. The dissipation due to the permeation and the sliding film can be written as

1 $$2\pi \eta {(\frac{R\dot{R}}{{R}_{L}\xi })}^{2}{R}_{L}{\xi }^{2}+k{\int }_{{R}_{L}}^{R}2\pi r{(\frac{R\dot{R}}{r})}^{2}dr=2\pi \eta (\frac{R{\dot{R}}^{2}}{{R}_{L}})+2\pi k{R}^{2}{\dot{R}}^{2}ln\frac{R}{{R}_{L}}$$

where *R* is the radius of the precursor film, *R*_*L*_ is the radius of the contact line between the aggregate and the precursor film which is nearly equal to the aggregate radius *R*_*0*_, $R\dot{R}/{R}_{L}$ is the velocity at the contact radius *R*_*L*_, *η* is the tissue viscosity, *k* is the friction coefficient of the cell aggregate with the substrate, and *ξ* is the width of the permeation region. The permeation is dominant if *η /R*_*L*_ > *k ln(R/R*_*L*_). This is the case of cell aggregates when the bulk viscosity *η* is much higher than the sliding viscosity^5^. The balance between the friction force *f*_*V*_ deduced from Eq. [1] ($dissipation=2\pi R\dot{R}{f}_{v}$) and the driving force *S* leads to:

2 $${\rm{\eta }}\frac{R\dot{R}}{{R}_{0}}=S\Rightarrow \frac{A-{A}_{0}}{{R}_{0}}={V}^{* }t$$

where *A* is the spread area and *V* ^*^ = *2π S/η is* the typical spreading velocity. The law of spreading is diffusive, with a diffusion coefficient *D* = *V* ^*^ *R*_0_ proportional to the radius of the aggregate and to the velocity *V* ^*^. Our aim is to investigate how the presence of particles changes the wetting behavior of the cellular aggregate.

## Results

### Preparation and characterization of MP-covered substrates

Fibronectin-coated glass slides were placed in the observation chamber of an optical microscope and immersed in cell culture medium. After a few minutes, MP suspensions of different concentrations were added to the medium and allowed to settle on the glass substrate. The sedimentation of MPs dispersed in water is characterized by the sedimentation length ${l}_{S}=\frac{{k}_{B}T}{{v}_{p}(\rho -{\rho }_{{H}_{2}O})g}$, where *k*_*B*_ *T* is the thermal energy, *v*_*p*_ the MP volume $({v}_{p}=\frac{\pi }{6}{d}^{3})$, *g* the gravitational acceleration, *ρ* the density of MPs and ${\rho }_{{H}_{2}O}$ the density of water. The values of *l*_*S*_ for each type of MPs are given in Table S1. If *l*_*S*_ is smaller than the MP size, (e.g. the case of SiO_2_CO_2_H), all MPs fall to the bottom of the observation chamber and the surface density *v* of sedimented particles is *v* = *CH*, where *C* is the particle concentration in the suspension and *H* is the height of the observation chamber, typically 4 mm. The corresponding surface fraction is ${\varphi }_{S}=\nu \pi {d}^{2}/4$. In the case of *l*_*S*_ larger than *d*, the MP surface concentration is smaller than predicted by the previous expression and must be measured optically. Substrates with MP surface fraction *ϕ*_*S*_ ranging from 10^−2^ to 1.5 were prepared by adjusting the concentration of the initial MP suspension. In the case of heavy particles, values of *ϕ*_*S*_ > 1 were attained, corresponding to several layers of MPs deposited on the substrate.

### Observation of aggregates spreading on MP-coated substrates

**Figure 1:**
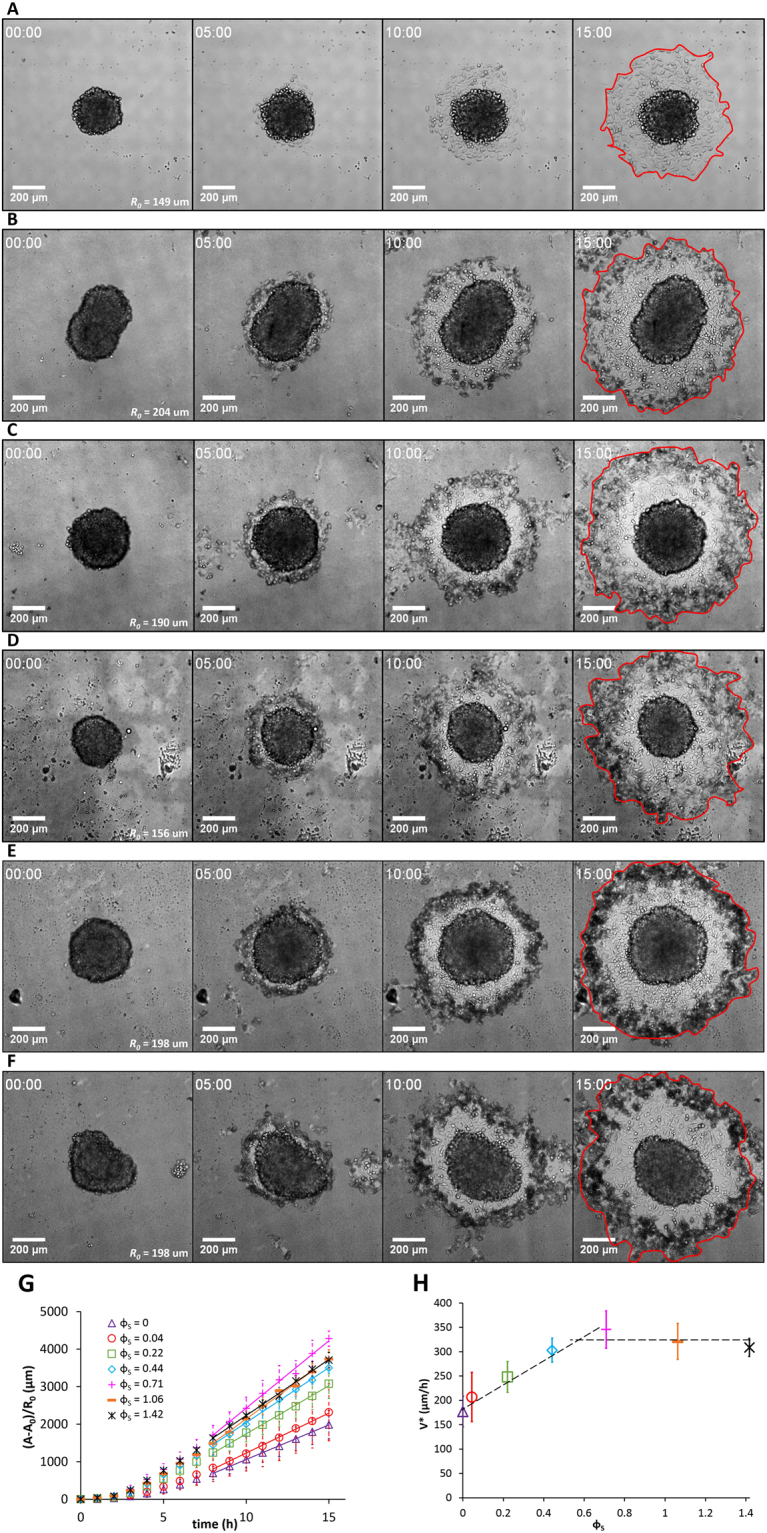
Spreading of ECad-GFP cell aggregates on glass substrates coated with fibronectin and decorated with SiO_2_Carbo1000 MPs. Surface fractions of MPs are (**A**) *ϕ*_*S*_ = 0.04, (**B**) *ϕ*_*S*_ = 0.22, (**C**) *ϕ*_*S*_ = 0.44, (**D**) *ϕ*_*S*_ = 0.71, (**E**) *ϕ*_*S*_ = 1.06, and (**F**) *ϕ*_*S*_ = 1.42. Aggregates are observed in bright field at *t* = 0, 5, 10, and 15 hours. (**G**) Time evolution of the monolayer area of spreading aggregates normalized by the initial aggregates radius *R*_0_ without particles and for various *ϕ*_*S*_. (**H**) Evolution of the spreading velocity of aggregates as a function of *ϕ*_*S*_. Error bars in (**G**) and (**H**) are standard deviations with *n* = 48, 9, 11, 11, 8, and 10 for *ϕ*_*S*_ = 0, 0.04, 0.22, 0.44, 0.71, and 1.06 respectively.

**Figure 2:**
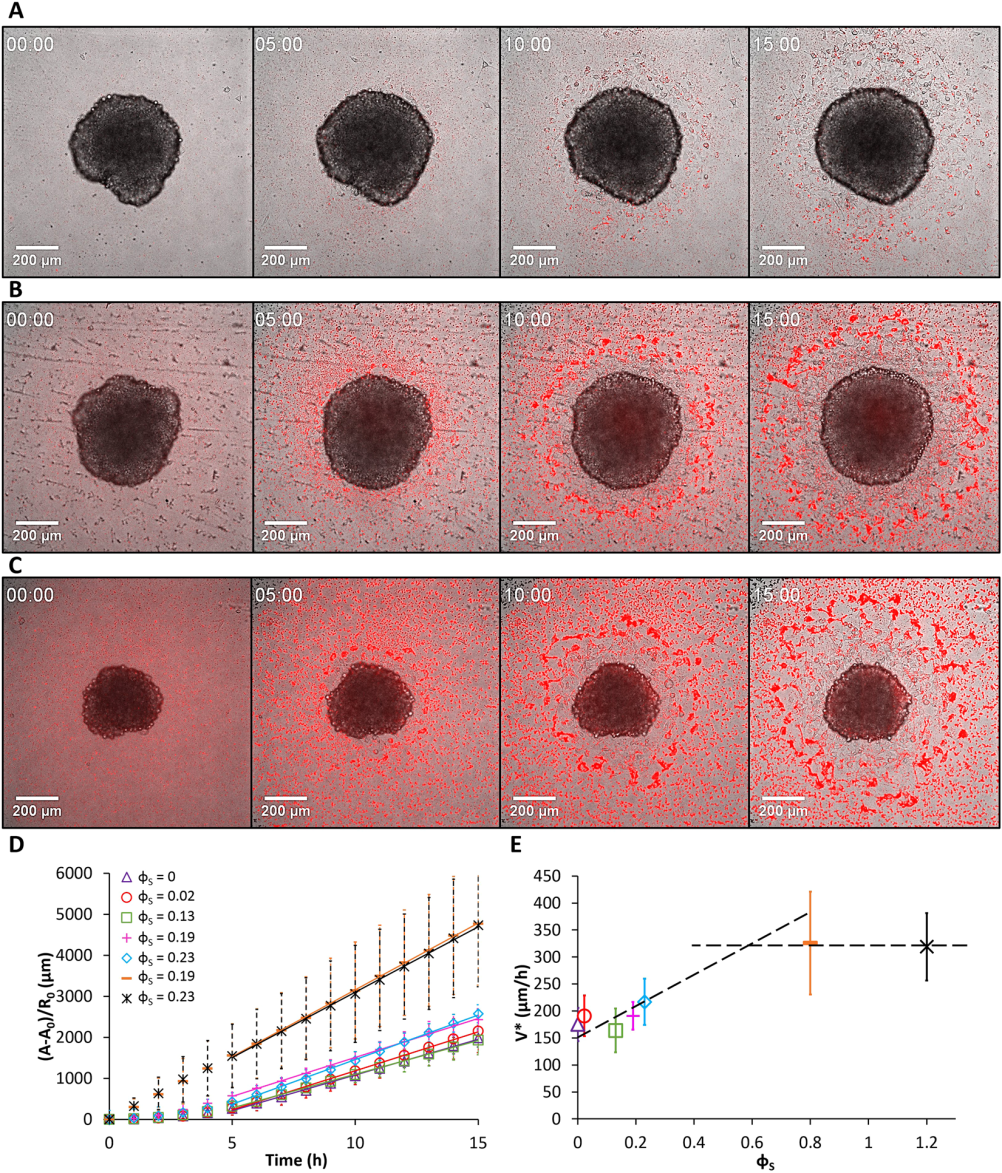
Spreading of ECad-GFP cell aggregates on glass substrates coated with fibronectin and decorated with fluorescent PsCarbo1000 MPs. Surface fractions of MPs are (**A**) *ϕ*_*S*_ = 0.02, (**B**) *ϕ*_*S*_ = 0.13, and (**C**) *ϕ*_*S*_ = 0.23. Aggregates are observed in bright field at *t* = 0, 5, 10, and 15 hours. (**D**) Time evolution of the monolayer area of spreading aggregates normalized by the initial aggregates radius *R*_0_ without particles and for various *ϕ*_*S*_. (**E**) Evolution of the spreading velocity of aggregates as a function of *ϕ*_*S*_. Error bars in (**D**) and (**E**) are standard deviations with *n* = 48, 55, 39, 5, 43, 9, and 11 for *ϕ*_*S*_ = 0, 0.02, 0.13, 0.19, 0.23, 0.8 and 1.2 respectively.

**Figure 3:**
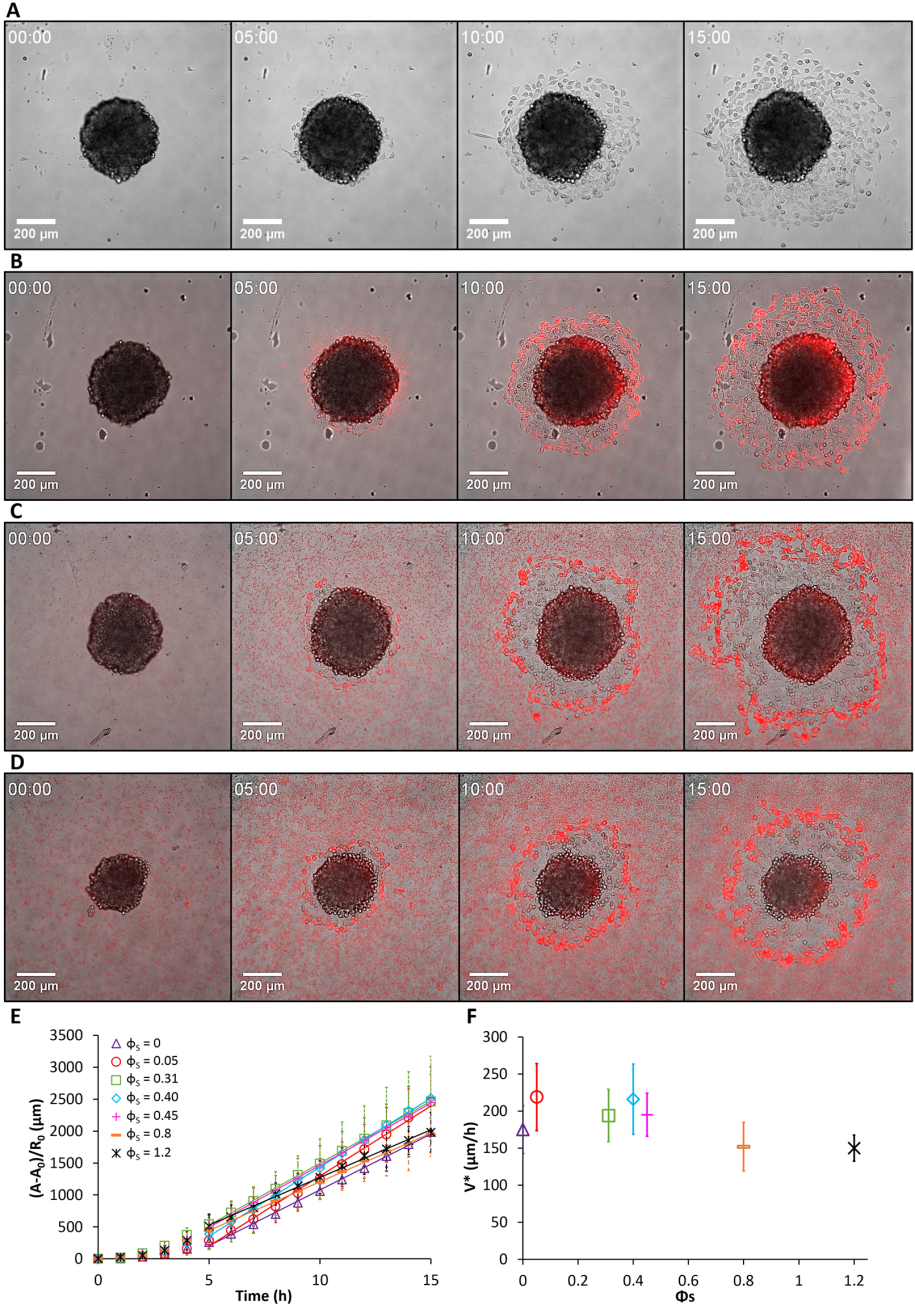
Spreading of ECad-GFP cell aggregates on glass substrates coated with fibronectin and decorated with fluorescent PsAmine200 MPs. Surface fractions of MPs are (**A**) *ϕ*_*S*_ = 0, (**B**) *ϕ*_*S*_ = 0.05, (**C**) *ϕ*_*S*_ = 0.40, and (**D**) *ϕ*_*S*_ = 0.45. Aggregates are observed in bright field at *t* = 0, 5, 10, and 15 hours. (**E**) Time evolution of the monolayer area of spreading aggregates normalized by the initial aggregates radius *R*_0_ without particles and for various *ϕ*_*S*_. (**F**) Evolution of the spreading velocity of aggregates as a function of *ϕ*_*S*_. Error bars in (**E**) and (**F**) are standard deviations with *n* = 48, 8, 44, 49, 51, 12, and 8 for increasing *ϕ*_*S*_.

**Figure 4:**
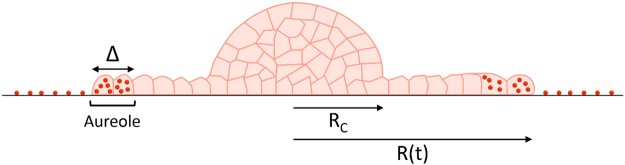
Schematic view of an aggregate spreading on a substrate coated with particles. The precursor film is a cell monolayer. Cells on the film periphery ingest the particles, forming an aureole of cells full of particles.

**Figure 5:**
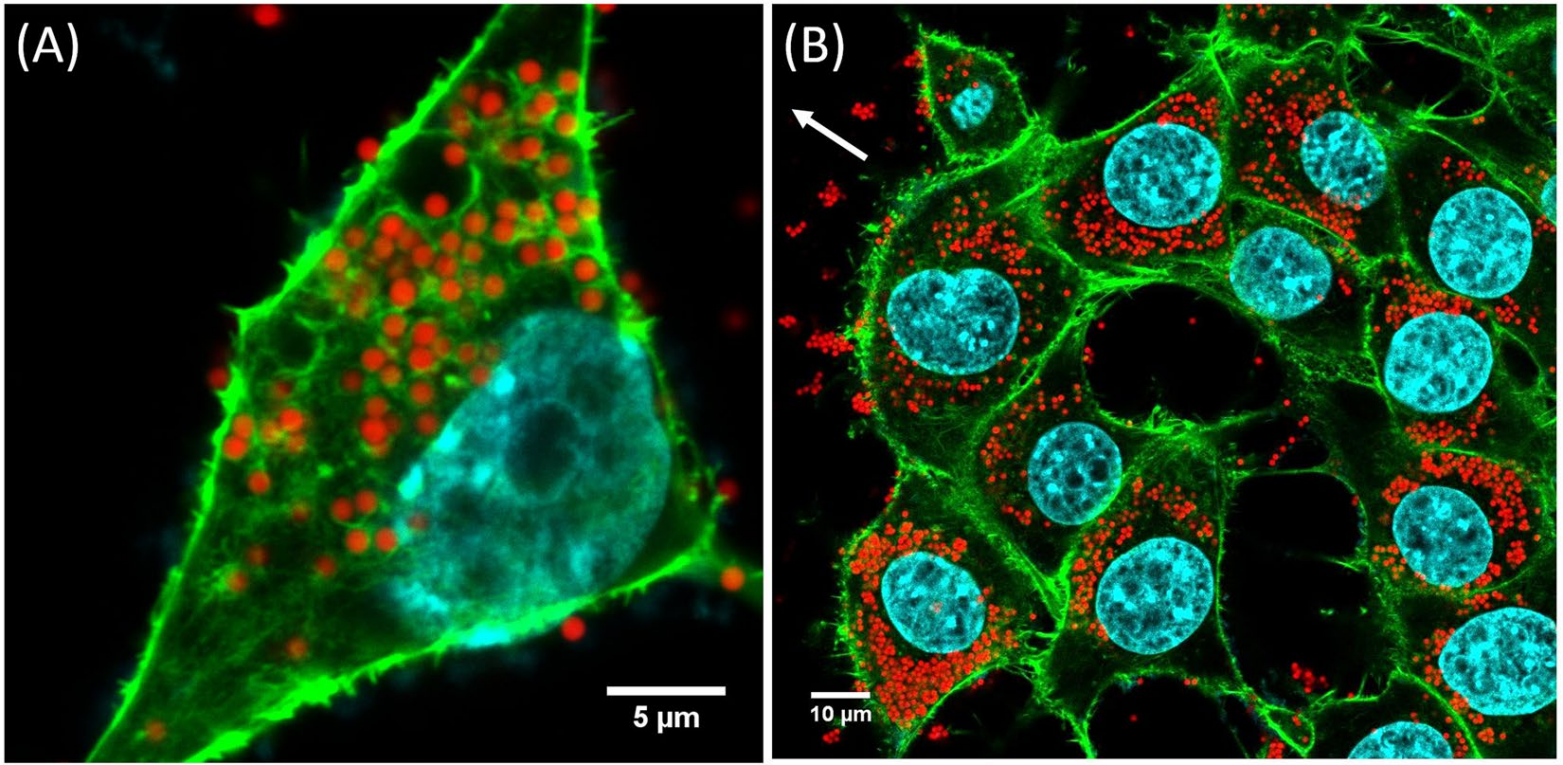
Internalization of MPs. Confocal micrographs of (**A**) a cell and (**B**) a group of cells on the film periphery of cellular aggregates spreading on carpets of PsCarbo1000 MPs (*ϕ*_*S*_ = 0.2) after 20 hours. The cytoskeleton and E-Cadherin are in green, the nucleus are stained in blue, and the fluorescence of the beads is red. The white arrow indicates the spreading direction.

Cellular aggregates, ~150 µm in size, were introduced into the chamber. Their spreading on the substrate was visualized by bright field microscopy and epifluorescence (see Figs 1 to 3). We present in Fig. 1 micrographs of aggregates deposited on SiO_2_Carbo1000-coated substrates with MP surface density ranging from 0.04 (top row) to 1.42 (bottom row). Micrographs on each row display the evolution of the aggregate from time = 0 (left) to time = 15 h (right). Some general features emerge and are represented schematically in Fig. 4: (1) the radius, *R*, of the precursor film increases with time, (2) an aureole of darker cells appears on the periphery of the precursor film, and (3) the area of the precursor film inside the aureole appears much brighter than the overall substrate coating, suggesting that the MPs on the substrate are internalized by the leading cells. Similar trends were observed for aggregates deposited on substrates covered with PSCarbo1000 (Fig. 2) or PSAmine200 (Fig. 3). The aureoles surrounding the precursor films are visualized by the red emission of the labeled MPs. To confirm that the MPs are internalized by the leading cells, we observed cells on the periphery of the precursor film by confocal fluorescence microscopy. Confocal micrographs of cells on the periphery of aggregates deposited on PsCarb1000-treated substrates are presented in Fig. 5 (see also Figure S2). The cells cytoskeleton and E-cadherin were stained in green and the nucleus was stained in blue. The distinctively red-emitting MPs appear throughout the cytoplasm of the cells. A few MPs are seen adsorbed on the outer cell membrane.

### Dynamics of the aggregate spreading as a function of the MP surface density

The radius *R* and the spreading area *A* of the precursor film were determined as a function of time and MP surface density for the three types of MPs. Plots of the spreading area *A* versus time are presented in Figs 1G, 2D, and 3E, for SiO_2_Carbo1000, PsCarbo1000, and PsAmine200, respectively. The *A* versus time relationship is linear in all cases, in agreement with Eq. [2]. The spreading velocity values, V*, were estimated from this relationship and plotted as a function of MP surface density values (Figs 1H, 2E, and 3F for the three types of MPs and Videos S1–S3). For *SiO*_2_ *Carbo1000* (Fig. 1H) and *PsCarbo1000* (Fig. 2E), *V* ^*^ increases with *ϕ*_*S*_ up to a certain value of *ϕ*_*S*_ and remains constant upon further increase of *ϕ*_*S*_. For *SiO*_2_ *Carbo1000*, *V* ^*^ increases from (4.9 ± 0.9) × 10^−2^ to *V* ^*^ = (9.6 ± 1.1) × 10^−2^ µm/s, the plateau value is attained for *ϕ*_*S*_ = 0.6. For *PsCarbo1000*, the spreading velocity *V* ^*^ versus *ϕ*_*S*_ increases up to a plateau value *V* ^*^ = (8.8 ± 1.7) × 10^−2^ µm/s corresponding to ${\varphi }_{S}^{P}=0.6$. The increase of the velocity *V* ^*^ is related to the increase in substrate roughness due to MPs deposition. An increase of the cell-substrate interface leads to an increase in the spreading parameter *S*, which can be written as ${V}^{\ast }({\varphi }_{S})={V}^{\ast }(0)\,(1+\alpha {\varphi }_{S})$, where *αϕ*_*S*_ is the excess area. Fitting the experimental data leads to *α* = 1.3 ± 0.2. The plateau value ${\varphi }_{S}^{P}=0.6$ corresponds to a dense packing of the spheres. In the case of *PsAmine200* MPs (Fig. 4F), *V* ^*^ shows no clear trend as a function of increasing *ϕ*_*S*_. It remains constant *V* ^*^ = (5.9 ± 2.0) × 10^−2^ µm/s. Suspensions of PsAmine200 in cell medium tend to flocculate when the MP concentration increases, as shown in Fig. S3 (see values in Table S1). The formation and deposition of flocs on the substrate may explain the different behavior of *PsAmine200* MPs, compared to the carboxylated MPs *SiO*_*2*_ *Carbo100* and *PsCarbo1000*.

### Collective uptake of MPs by leading cells

**Figure 6:**
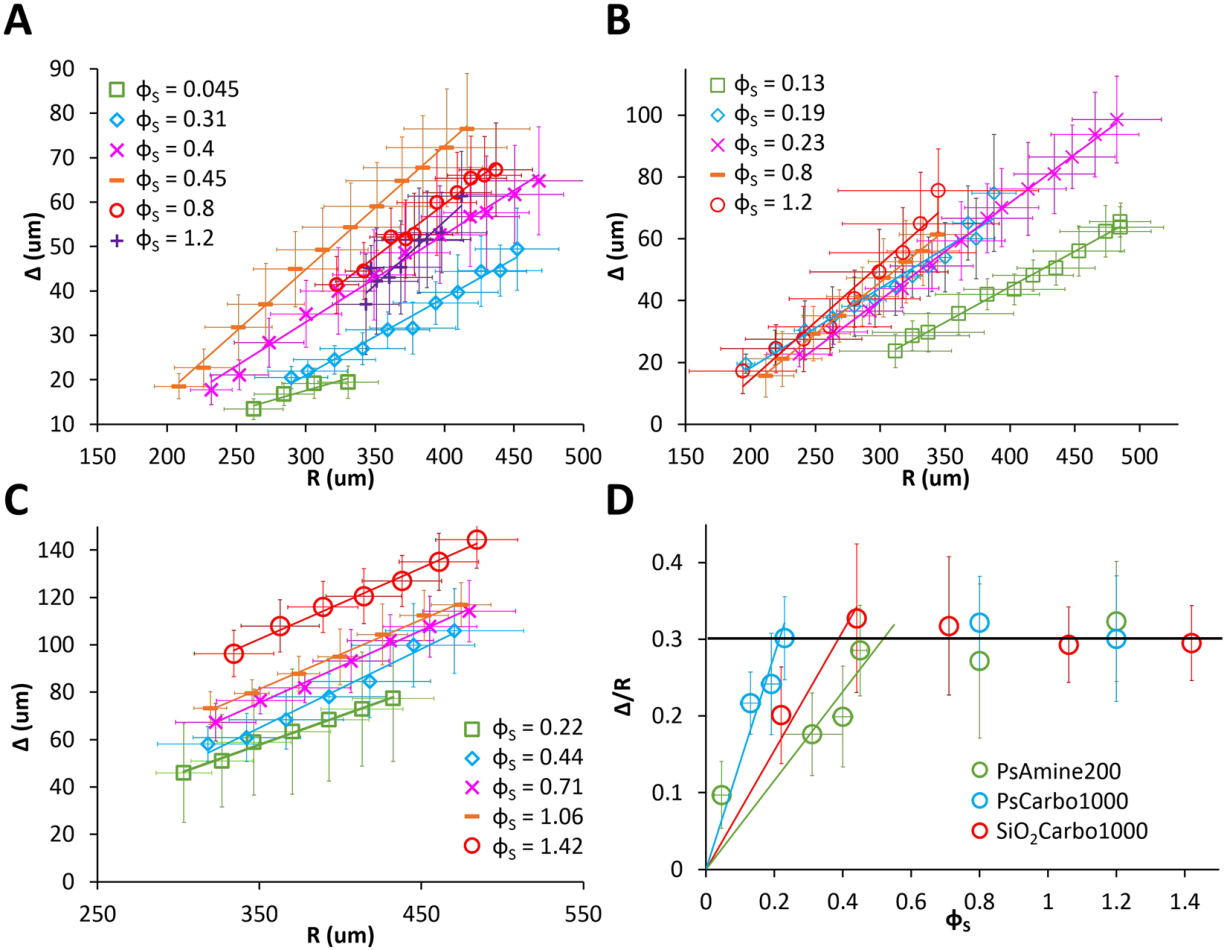
Thickness of the aureole. (**A–C**) Evolution of the thickness of the black aureole, *Δ*, versus the radius of the spreading film *R* for PsAmine200, PsCarbo1000, and SiO_2_Carbo1000 MPs respectively. (**D**) Representation of *Δ/R* as a function of the MPs density *ϕ*_*S*_. Blue, green, and red lines in (**D**) are fits to the experimental points using Eq. [5]. The black line in D is a guide to the eye. Error bars are standard deviations with *n* ≥ 10.

As seen qualitatively in the optical micrographs shown in Figs 1, 2, and 3, the migrating cells on the periphery of the precursor film internalize the MPs that littered the substrate. The MP concentration inside the cells, *C*_*i*_, increases up to a concentration *C*_*is*_, for which the leading cells are saturated in MPs. We call *R*_*c*_, the film radius corresponding to *C*_*is*_, At this point, the cells immediately following the leading cells start to take up MPs left behind by the leaders. The widths, *Δ*, of the aureoles defined in Fig. 4, are determined from image analysis of Figs 1–3 (see Methods). The experimental values of *Δ* are plotted in Fig. 6A–C as a function of the radius *R* for the three types of MPs. Measurements of cell darkness (see Fig. S1) indicate that the MPs density inside the aureole is constant. *Δ* increases linearly with *R* and with the density *ϕ*_*S*_ of MPs. For large *ϕ*_*S*_ the darkness of the aureole (Fig. S1) is very pronounced, which we take as an indication that the MPs are not only internalized by cells but also adsorbed on the cell membrane as observed in Fig S2.

The formation of the aureole in the case of low MP concentration and its extension at long times in the case of all MP concentrations are analyzed by a model used successfully in our previous studies of the cell internalization of PsCarbo, PsAmine, and silica nanoparticles (NP)^6^. We demonstrated that as one increases the NP concentration in a suspension of cells, the NPs are internalized by the cells up to a concentration *C*_*is*._, above which, NPs adsorb on the cell’s membrane.

*Δ* can be derived from the volume conservation of MPs, which are either internalized by cells or adsorbed on the cell membrane (above a threshold *ϕ*_s_^*^). The number of MPs in the aureole is equal to the number of MPs that have been cleared from the substrate, leading to:

3 $$\frac{2\pi R{\rm{\Delta }}}{{A}_{cell}}({C}_{i}{V}_{cell}+2{C}_{S}{A}_{cell})=\pi {R}^{2}\nu $$

where *C*_*i*_ is the MP concentration inside the cell, *C*_*S*_ the MP surface concentration on the cell membrane, *A*_*cell*_ the projected cell area, and *V*_*cell*_ the volume of the cell.

For the first row of cells *Δ* = *d*_*cell*_ (=22 μm, as determined experimentally, see Methods), *C*_*i*_ increases up to *C*_*is*_, which defines the radius *R*_*c*_ of the aureole above which the leading cells are full of particles. *R*_*c*_ is therefore given by Eq. [3] with *Δ* = *d*_*cell*_, *Cs* = 0, *C*_*i*_ = *C*_*is*_. It may be written as:

4 $${R}_{c}{\varphi }_{s}=3{\phi }_{is}\frac{{d}_{cell}}{d}\frac{{V}_{cell}}{{A}_{cell}}$$

where *ϕ*_*is*_ = *C*_*is*_ *v*_*p*_ is the maximum volume fraction occupied by the MPs in the cell.

For *d* = 1 µm, *V*_*cell*_ = 1600 µm^3^, *A*_*cell*_ = 770 µm^2^, ${R}_{c}{\varphi }_{s}=137{\phi }_{is}$ µm. A simple measurement of *R*_*c*_ for a given *ϕ*_*S*_ gives a straightforward approximation of *φ*_*is*_. Typically, *R*_*c*_ ~ 106 µm for *ϕ*_*S*_ = 0.22, leading to *φ*_*is*_ = 0.17.

For *R* larger than *R*_*c*_, Eq. [3] with *φ*_*i*_ = *φ*_*is*_ leads to:

5 $${\rm{\Delta }}(3\frac{{\phi }_{is}}{d}\frac{{V}_{cell}}{{A}_{cell}}+4{\phi }_{s})=R{\varphi }_{s}$$

where *φ*_*s*_ is the MPs surface fraction adsorbed on the cell membrane.

Equation [5] indicates that *Δ* increases linearly with *R*, as observed experimentally. Plots of *Δ/R* versus *ϕ*_*S*_ are presented in Fig. 6D. For small *ϕ*_*S*_ ($\ll$ *ϕ*_*S0*_, see Table 1), Δ/R increases linearly and all particles are internalized. A fit of the data with Eq. [5] with *φ*_*s*_ = 0 leads to value of *φ*_*is*_ for each type of MPs. For larger *ϕ*_*S*_ values, *Δ/R* values reach a plateau, implying that MPs are not only internalized, but are also adsorbed on the cells membrane. The plateau of *Δ/R* versus *ϕ*_*S*_ leads to a linear relationship between *φ*_*S*_ and *ϕ*_*S*_ above a threshold value, *ϕ*_*S0*_. The data are fitted by *φ*_*S*_ = *a*(*ϕ*_*S*_ − *ϕ*_*S*0_). The plateau value *Δ/R* = 0.3 is the same for all types of MPs, leading to *a* = 0.9. We give in Table 1 the values of *φ*_*is*_ and *φ*_*S*_ versus *ϕ*_*S*_ for the 3 types of MPs. We also give the total number of particles internalized, *n*_*i*,_ and adsorbed, *n*_*s*_, by each cell. *n* = *n*_*i*_ + *n*_*s*_ represents the total number of particles captured by each cell in the aureole. Using our model, we find *φ*_*is*_ values for MPs a hundred times larger than the *φ*_*is*_ values previously obtained for NPs^7^. MPs occupy a cell volume fraction on the order of 0.1 (see Table 2), a value close to the volume fraction occupied by the cell nucleus (0.25) (see Table 2).

**Table 1:** Parameters φ_is_ and φ_s_ for all tested types of particles.

Particles	*ϕ*_*S*_	φ_is_	*φ*_*s*_	*ϕ*_*S0*_	*n*_*i*_^[a]^	*n*_*s*_^[a]^	*n*_*tot*_^[a]^
PsAmine200	0.05	0.23	0	~ 0.5	1150	0	1160
	0.31	0.23	0	~ 0.5	1150	0	1160
	0.40	0.23	0	~ 0.5	1150	0	1160
	0.45	0.23	0	~ 0.5	1150	0	1160
	0.8	0.23	0.28	~ 0.5	1150	540	1700
	1.2	0.23	0.47	~ 0.5	1150	920	2070
PsCarbo1000	0.13	0.11	0	~ 0.2	330	0	330
	0.19	0.11	0	~ 0.2	330	0	330
	0.23	0.11	0	~ 0.2	330	0	330
	0.8	0.11	0.43	~ 0.2	330	830	1160
	1.2	0.11	0.80	~ 0.2	330	1550	1880
SiO_2_Carbo1000	0.22	0.21	0	~ 0.4	630	0	630
	0.44	0.21	0	~ 0.4	630	0	630
	0.71	0.21	0.22	~ 0.4	630	460	1090
	1.06	0.21	0.57	~ 0.4	630	1120	1750
	1.42	0.21	0.87	~ 0.4	630	1700	2330

^a^It corresponds to the number of clusters of particles in the case of PsAmine200 particles.

**Table 2:** Cell volume fraction of the nucleus and *φ*_*is*_ internalized by the cells at the periphery of the film, obtained from analysis of confocal micrographs.

Particles	Volume fraction of beads	Volume fraction of the nucleus
PsAmine200 (*n* = 6)	0.11 ± 0.04	0.28 ± 0.05
PsCarbo1000 (*n* = 10)	0.12 ± 0.05	0.24 ± 0.05

We confirmed the validity of these estimates by confocal microscopy. Z-stack micrographs of cells on the periphery of the precursor film spreading around aggregates deposited on a substrate with PsCarbo1000 and PsAmine200 (*ϕ*_*s*_ = 0.2) were taken every 0.25 µm (Figure S2). We calculate the volumes of the beads, of the nuclei, and of the cells from the Z-stack micrographs separated by 0.25 µm. The volumes are calculated by summing elementary volumes between two successive images defined as the area (*A*_*bead*_, *A*_*nucleus*_, and *A*_*cell*_, respectively) times 0.25 µm. In the case of amine200 MPs, there is a discrepancy by a factor of two between the volume fraction of beads obtained using the spreading experiment with our model and the volume fraction of beads obtained from the confocal experiments. In contrast, the two values are in good agreement for PsCarbo1000. These results are attributed to differences in the stability of PsCarbo1000 and PsAmine200 suspensions in cell culture medium. PsAmine particles flocculate and form large aggregates, which cannot be internalized and may be adsorbed on the cells (see Figure S4). The PsCarbo1000 suspensions are stable, with no sign of flocculation upon standing.

The volume fraction of MPs found inside the cells on the film periphery is consistent with the mechanism of phagocytosis that controls MPs internalization. The engulfment of MPs by the cell membrane involves actin polymerization^8,9^. The characteristic internalization time depends on the shape, size, and surface chemistry of the MPs. For instance, the internalization time of silica microspheres increases from 1.5 to 4.3 minutes when their diameter increases from 1.85 to 3 µm^10^. Similar internalization times are measured for latex microspheres (2.5 min for MPs with a 2 µm diameter)^10^. Cells can internalize beads as large as 5 µm by this mechanism. The cell volume fraction occupied by such beads can be very large, 10% for a cell engulfing three 5-µm beads^10^. Unlike MPs, NPs enter cells by pinocytosis:^10^ they are internalized in endosomes through a rapid and saturable clathrin-mediated process with a characteristic time τ ≈ 2 min^11,12^. The maximal volume fraction of the cell occupied by the NPs is on order 2 × 10^−3^ regardless of the particle size^7,13^.

## Discussion

We have observed the clearing of particles by migrating cells during the spreading of cellular aggregates. MPs are taken up by cells on the periphery of the precursor film expanding outwards from an aggregate. When cells of the outermost row of the film are full, cells from the second row take over, internalizing the MPs that remained on the substrate. This leads to the formation of an aureole of cells full of MPs around a film cleared of MPs. For low MP surface density, all particles are internalized by the migrating cells until the MP volume fraction inside the cells reaches a value ranging from 0.1 to 0.2. This value is one hundred times larger than the volume fraction occupied by NPs endocytosed by cells. When the MP surface density is much higher, particles are internalized by the cells and also adsorbed on the cell membrane.

This work leads to a new method to quantify the uptake of MPs by cells. The clearing of MPs requires that the internalization time *τ*_*i*_ is smaller than the passage time *τ*_*p*_ defined by ${V}^{\ast }{\tau }_{p}={d}_{cell}$. With *d*_*cell*_ = 22 µm and *V* ^*^ = 6.2 × 10^−2^ µm/s, we find *τ*_*p*_ = 6 min. This time is larger than the internalization time of MPs of size 1 μm (~ 2 min). Particles larger than ~5 μm are not internalized as *τ*_*i*_ exceeds *τ*_*p*_. In preliminary studies, we observed that such large MPs are pushed away by the spreading film. Activated by the cells through energy transfer during collisions, the MPs then behave like Brownian particles. The physics is completely different and will be discussed in a separate paper. As it has been shown that the internalization time of soft beads is much longer than in the case of rigid beads, it will also be interesting to expand our studies to this case in order to quantify the role of particle rigidity on phagocytosis^14^. We also plan to study how cells divide when a large fraction of their volume is occupied by MPs. In Fig. 5B we see a cell division where all the particles are pushed to the cell periphery. Whereas the role of nanoparticles, which occupy a very small fraction of the cell volume, on the cell cycle is well studied, the effect of large beads is still unknown and may have implications in cancer proliferation.

The clearing of particles discussed here also plays a role in wound healing where macrophages and leucocytes clean wounds by elimination of dead cells, germs, and bacteria^15^. Another example is the clearing of lungs from inhaled carbon or mineral particles^8^. Finally, the collective clearing of MPs by migrating cells bears similarities with the phenomenon of autophagy, the self-eating of cells elucidated by Oshumi, who was awarded the Physiology Nobel prize in 2016. Striking parallels exist between the observations reported here and the phagocytosis of cell debris by the spreading precursor film we detected recently (Video S4). In this case too, migrating cells cleaned the substrate of all debris as they spread around the aggregate.

## Methods

### Materials

Fibronectin and phosphate-buffered saline (PBS) were purchased from Sigma-Aldrich Co. Trypsin-EDTA, penicillin-streptomycin, Dulbecco’s Modified Eagle Medium (DMEM), and the fluorescently labeled polystyrene particles (FluoSpheres®) were obtained from Life Technologies Co. Silica particles (unlabeled) were obtained from Corpuscular Co.

### Preparation of coated glass substrates

Circular glass coverslips (Diameter: 25 mm) were cleaned by sonication in ethanol (5 min) followed by drying at room temperature, and exposure to deep UV for 5 min. They were coated with a solution of fibronectin (0.1 mg/mL) in PBS (pH 7.4) for 1 h at room temperature. The coverslips were rinsed with PBS and used immediately.

### Cell culture and aggregates preparation

Ecad-GFP cells were used throughout the study. They are murine sarcoma S180 cells stably transfected to express E-cadherin-GFP^16^. They were cultured at 37 °C under 95% air/5% CO2 atmosphere in a culture medium consisting of Dulbecco’s Modified Eagle Medium (DMEM) supplemented with 10% (vol/vol) Fetal Bovine Serum (FBS) and antibiotics (100 μm/mL streptomycin and 100 U/mL penicillin). The average volume (*V*_*cell*_ = 1620 µm^3^) of the cells was measured in the medium where they are spherical, before they spread on the substrate, and was estimated by measuring 100 cells. The average diameter of the first row of cells (*d*_*cell*_ = 22 µm) in the spreading film was measured 15 h after aggregates have been deposited on a substrate, and was estimated by measuring 100 cells.

Upon reaching confluence, the cells were collected and treated for aggregation by the hanging droplet method^17^.

### Aggregate spreading and imaging by bright field microscopy

A specific volume of a dispersion of the MPs in the medium of desired concentration, was placed on a fibronectin-coated glass coverslip fitted in a magnetic imaging chamber (Chamlide CMB, CM-B25-1) filled with CO_2_-equilibrated culture medium. After a given time, ranging from few minutes for silica particles and after a few hours for polystyrene particles, aggregates were deposited at random on the coated glass coverslip. The final volume of cultured medium was 1.5 mL. The chamber was sealed with mineral oil to prevent water evaporation and placed for viewing in an inverted microscope (TIRF AF 6000LX, Leica) equipped with a ×10 0.30 NA objective. Videos were recorded with a CCD camera (Photometrics Cascade 512B, Roper Scientific) at an acquisition rate of 1 frame every 10 min. Images were exported from the instrument software in TIFF format and visualized using the ImageJ software package v.1.46r (National Institutes of Health, Bethesda, MD).

### Image analysis

The areas of the precursor films, A, were obtained by tracing by hand the contour (perimeter) of the films with ImageJ on bright field micrographs for all the types of particles. The values of A_0_ were used to calculate R_0_. We approximate aggregates as spheres to calculate R_0_.

The area of a cell (A_cell_) was determined by taking the average value of 100 apparent areas of spread cells in the aureole surrounding the precursor film. The MP surface concentrations ϕ_S_ have been measured optically using the Threshold option of the ImageJ software 15 h after placing the aggregate on the substrate.

The widths, Δ, of the aureoles were determined from image analysis using the ImageJ software (outer and inner perimeters of the aureoles). For polystyrene particles, merged micrographs (bright field and fluorescence) were used to determine the areas. At a given time, areas were measured by tracing the contours of the spreading aggregate with and without the aureole. These areas give the values of the mean radii of the precursor films with and without the aureole. The difference between these 2 radii gives the mean value of the widths of the aureole, *Δ*, at a given time.

The volume fractions of the nucleus and the beads in cells were determined by confocal microscopy. Z-stacks of cells were performed with a distance of 0.25 μm between 2 consecutive micrographs. A Z-stack of the cytoskeleton and E-Cadherin was used to determine the perimeter of the cells. Other ones were used for the nucleus and for the fluorescent beads. For each picture of each Z-stack we measured the area of the cell (A_cell_), the area of the nucleus (A_nucleus_), and the area of the internalized beads (A_bead_) using the threshold option of ImageJ. The volumes of the cells, nucleus, and internalized beads were calculated by summing elementary volumes between two successive images defined as the area (A_cell_ A_nucleus_, and A_bead_, respectively) times 0.25 µm. 6 cells were analyzed for PsAmine200 beads and 12 cells in the case of PsCarbo1000.

### Aggregate spreading and imaging by confocal microscopy

Aggregates were deposited on MP-treated fibronectin-coated glass coverslips prepared as described above in magnetic imaging chambers. The samples were incubated for 20 h at 37 °C under a 95% air/5% CO_2_ atmosphere). The aggregates were fixed and stained with Alexa Fluor® Phalloidin (cytoskeleton) and DAPI (nucleus) using a protocol described in SI. The stained aggregates were observed with an inverted confocal microscope (TCS SP5, Leica Microsystems) equipped with a ×63 1.4 NA objective with oil immersion. Images were exported from the instrument software in TIFF format and visualized using the ImageJ software package v.1.46r (National Institutes of Health, Bethesda, MD).

## Electronic supplementary material

Supporting Information

Spreading of ECad-GFP cell aggregates on glass substrates coated with fibronectin and decorated with SiO2Carbo1000 MPs

Spreading of ECad-GFP cell aggregates on glass substrates coated with fibronectin and decorated with fluorescent PsCarbo1000 MPs.

Spreading of ECad-GFP cell aggregates on glass substrates coated with fibronectin and decorated with fluorescent PsAmine200 MPs.

Phagocytosis of cell debris by the spreading precursor film of an aggregate
