# Supplementary material for: How gluttonous cell aggregates clear substrates coated with microparticles
Source: Sci Rep. 2017 Nov 16;7:15729. doi: 10.1038/s41598-017-15665-2 (PMC5691206; doi:10.1038/s41598-017-15665-2)
Supplement: Supplementary file 1 — Supporting Information [file 41598_2017_15665_MOESM1_ESM.pdf]

How gluttonous cell aggregates clear substrates coated with microparticles

Grégory Beaune[1], Andy Y.W. Lam[1], Sylvie Dufour[2,3], Françoise M. Winnik†[1,4] & Françoise Brochard-Wyart†*[5,6]*

Supporting Information

Fixing and staining of aggregates after incubation for 20 h on MP-treated substrates. Cells are fixed in 4% paraformaldehyde (PFA)-PBS for 15 min and then rinsed with PBS. Cells are permeabilized and blocked with a BSA-Triton solution (1% BSA, 0.1% Triton X-100 in PBS) on ice for 15 min and then rinsed 3 times for 5 min. The cytoskeleton of the cells is stained for 20 min with Alexa Fluor® Phalloidin (A12379, ThermoFisher Scientific) diluted in 1% BSA-PBS with a dilution of 1/15. The nucleus of the cells was stained for 1 h with DAPI in 1% BSA-PBS. Finally, the chamber is rinsed 3 times with PBS for 15 min on a shaker and following PBS removal, aggregates are protected from photobleaching using a slow antifade reagent in glycerol/PBS

Table S1. Hydrodynamic Diameter in cell culture medium with 10% FBS (0.1 % of particles v/v) and sedimentation length of the used particles.

Particles	TEM Diameter (nm) [a]	AF4 Diameter (nm) [b]	Sedimentation length l_S (µm)
PsCarbo	1100 ± 36	1004 ± 127	16
PsAmine	200 ± 10	840 ± 62	28
SiO_2Carbo	1000	-	0.5

[a] Provided by the supplier.

[b] Measured by asymmetrical flow field-flow fractionation (AF4) in cell culture medium supplemented with 10% FBS.

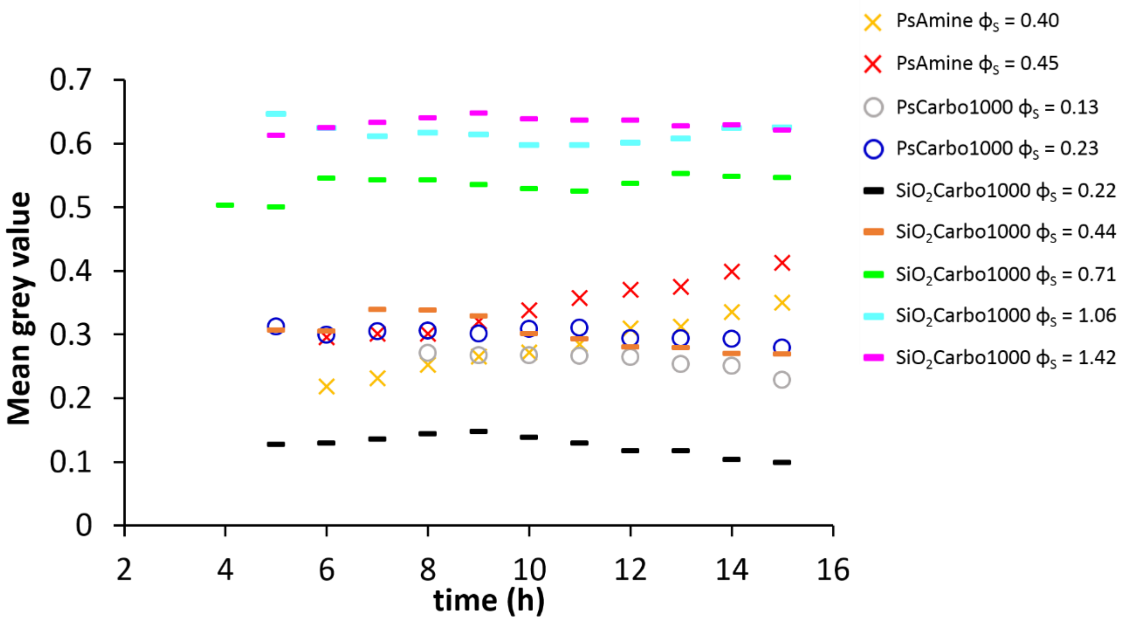

Figure S1. Evolution of the mean grey value of the aureoles as a function of the time.

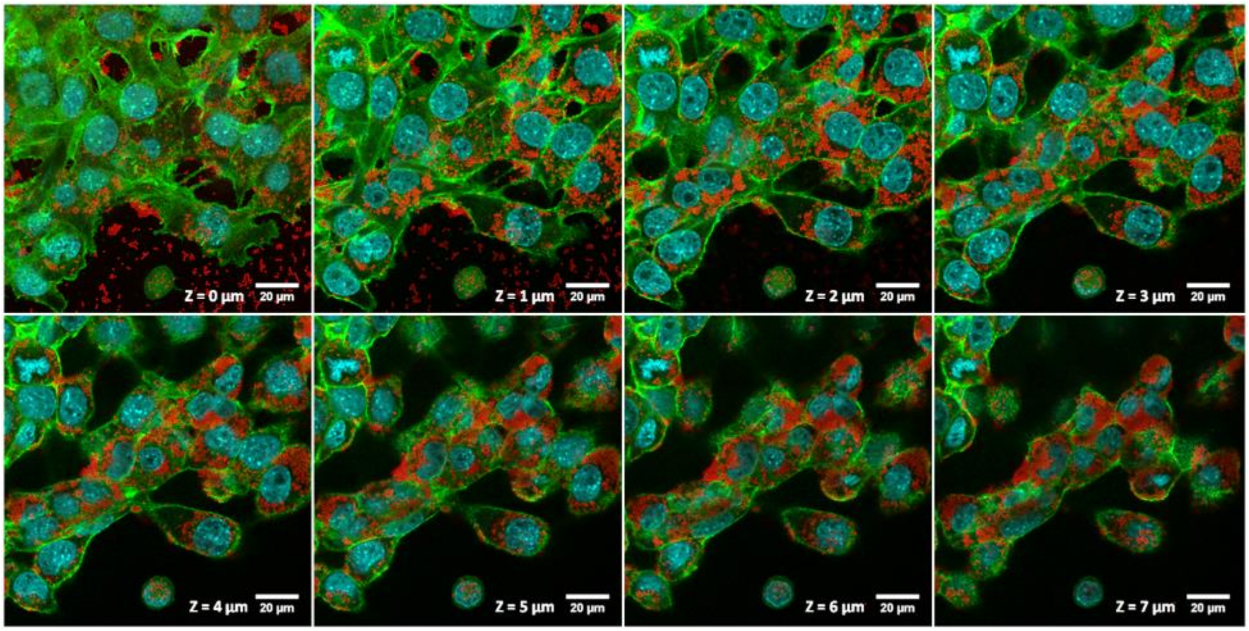

Figure S2. Internalization of MPs. Z stack confocal microscopy pictures at the film periphery of a cellular aggregate spreading on a carpet of PsCarbo1000 MPs after 20 hours. The cytoskeleton and E-Cadherin are in green, the nucleus is stained in blue, and the fluorescence of the beads is red.

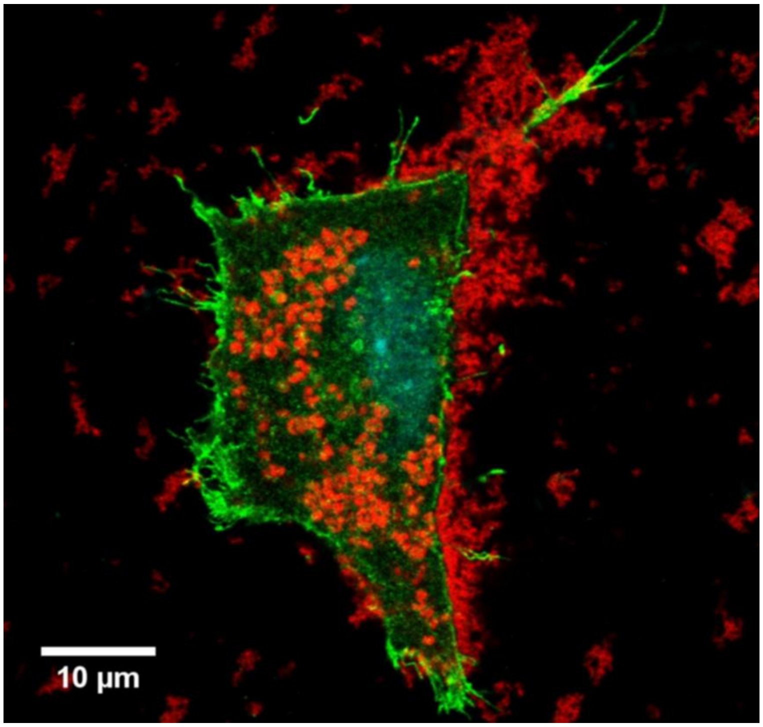

Figure S3. Confocal microscopy picture of a cell at the periphery of the film of a cellular aggregate spreading on a carpet of PsAmine200 MPs after 20 hours. The cytoskeleton and E-Cadherin are in green, the nucleus is stained in blue, and the fluorescence of the beads is red.

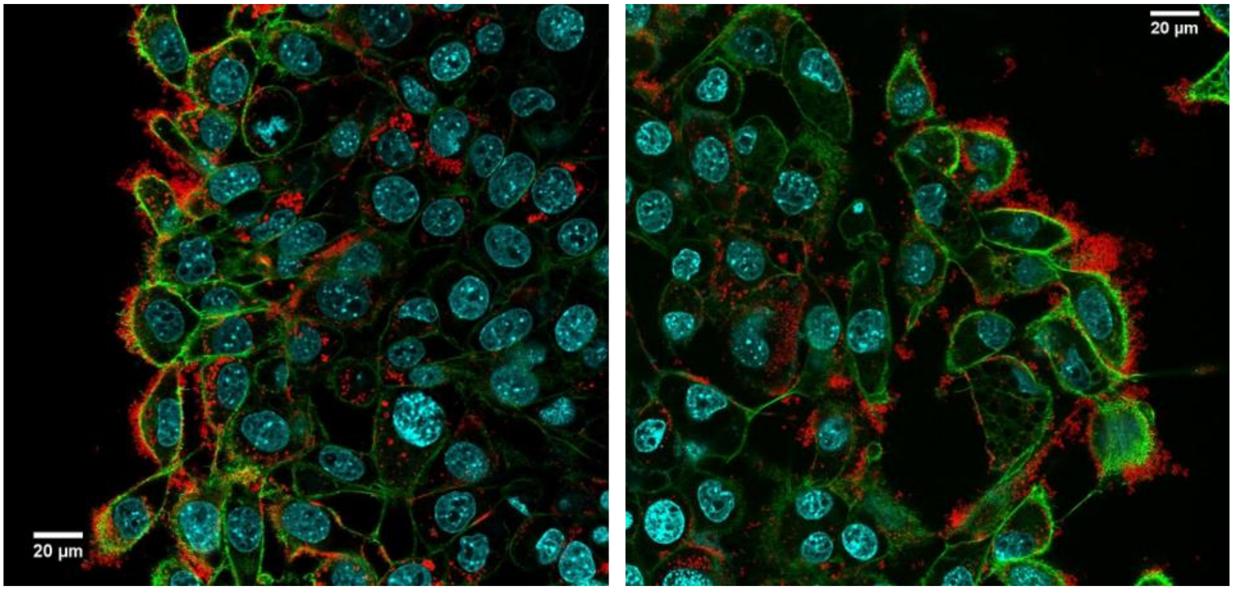

Figure S4. Confocal microscopy pictures of cells at the periphery of the film of cellular aggregate spreading on carpets of PsAmine200 MPs after 20 hours. The cytoskeleton and E-Cadherin are in green, the nucleus is stained in blue, and the fluorescence of the beads is red.

Glossary

d	Diameter of the particles
v_p	Volume of the particles
R	Radius of the precursor film
R_L	Radius of the contact line between the aggregate and the precursor film
R_0	Initial radius of the aggregate
η	Viscosity of the aggregates
ξ	Width of the permeation region
k	Friction coefficient of the cell aggregate with the substrate
A	Contact area between the aggregate and the substrate
V^*	Characteristic spreading velocity of the aggregate
l_S	Sedimentation length
$k_B T$	Thermal energy (Boltzmann constant and temperature)
ρ	Density of particles
ρ_{H_2O}	Density of water
C	Particle concentration in solution
H	Height of the observation chamber
ν	Density of particles on the substrate
ϕ_S	Surface fraction of particles on the substrate
C_i	Particle concentration inside the cells
C_{is}	Maximal particle concentration inside the cells
R_C	Radius of the precursor film corresponding to C_{is}
Δ	Widths of the aureole
C_S	Particle surface concentration on the cell membrane
A_{cell}	Projected cell area
V_{cell}	Cell volume
d_{cell}	Cell diameter
φ_{is}	Maximal volume fraction of particles inside the cells
φ_s	Surface fraction of particles on the cell membrane
n_i	Number of particles internalized by each cell

n_s	Number of particles adsorbed on the membrane of each cell
n	Total number of particles captured by each cell in the aureole
τ_i	Internalization time τ_i
τ_p	Passage time
